# Postmarketing surveillance on the clinical use of edoxaban in patients with nonvalvular atrial fibrillation (ETNA‐AF‐Japan): One‐year safety and effectiveness analyses

**DOI:** 10.1002/joa3.12332

**Published:** 2020-03-24

**Authors:** Takeshi Yamashita, Yukihiro Koretsune, Tomoko Nagao, Kazuhito Shiosakai

**Affiliations:** ^1^ The Cardiovascular Institute Tokyo Japan; ^2^ National Hospital Organization Osaka National Hospital Osaka Japan; ^3^ Post Marketing Study Department Daiichi Sankyo Co. Ltd. Tokyo Japan; ^4^ Biostatistics and Data Management Department Daiichi Sankyo Co. Ltd. Tokyo Japan

**Keywords:** anticoagulants, atrial fibrillation, DOAC, edoxaban, postmarketing surveillance

## Abstract

**Background:**

The safety and effectiveness of edoxaban in real‐world clinical settings have not yet been elucidated thoroughly among Japanese patients with nonvalvular atrial fibrillation (NVAF). We report the one‐year interim results of ETNA‐AF‐Japan (Edoxaban Treatment in routiNe clinical prActice in patients with nonvalvular Atrial Fibrillation: UMIN000017011), an ongoing two‐year study.

**Methods:**

ETNA‐AF‐Japan is a prospective, real‐world multicenter observational study that analyzes the long‐term safety and effectiveness of edoxaban. Physicians recorded clinical characteristics, bleeding events, and clinical events of ischemic stroke and systemic embolism, among others.

**Results:**

In total, 11 569 patients with NVAF were enrolled. The demographic and clinical characteristics of the patients in the safety analysis set (n = 11 107) were a mean age of 74.2 ± 10.0 years; female sex, 40.6%; age ≥75 years, 52.4%; body weight ≤60 kg, 54.3%; creatinine clearance ≤50 mL/min, 31.2%; mean CHADS_2_ score of 2.2 ± 1.3. The mean treatment period was 311.2 days (median; 366.0 days), and ~80% of patients continued edoxaban treatment. In the safety analysis, the incidence of all bleeding events was 6.32% [95% CI: 5.87, 6.79] (n = 702), and for major bleeding, it was 1.08% [0.90, 1.29] (n = 120). In the effectiveness analysis set (n = 11 059), the incidence of ischemic stroke (excluding TIA) or systemic embolism was 1.10% [0.92, 1.32] (n = 122).

**Conclusions:**

At one‐year follow‐up, the results showed no major concerns about the safety and effectiveness of edoxaban in Japanese patients with NVAF in a real‐world clinical setting.

## Graphical abstract

The safety and effectiveness of edoxaban in real‐world clinical settings have not yet been elucidated thoroughly among Japanese patients with nonvalvular atrial fibrillation (NVAF). We report the one‐year interim results of 11 107 patients in the ETNA‐AF‐Japan study, an ongoing two‐year postmarketing surveillance. The results showed no major concerns about safety and effectiveness of edoxaban in Japanese patients with NVAF in a real‐world clinical setting

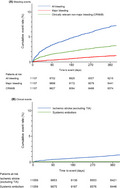

## 1 INTRODUCTION

In patients with nonvalvular atrial fibrillation (NVAF), treatment with a direct oral anticoagulant (DOAC)^1^, ^2^, ^3^, ^4^ for ischemic stroke prevention is now preferred over warfarin and especially considered for use in patients who are planning to start anticoagulant therapy as described in the “2013 Guidelines for Pharmacotherapy of Atrial Fibrillation” (JCS 2013) of the Japanese Circulation Society^5^, ^6^ and the 2018 European Heart Rhythm Association Practical Guide.^7^ However, the guides have pointed out that patients with atrial fibrillation (AF) typically have concomitant diseases and other risk factors, and their long‐term, day‐to‐day experience with the treatment warrants further investigation. A study of the long‐term use of a DOAC in a large sample‐size (≥10 000 patients) that analyzes the reduction in risk of ischemic stroke and systemic embolism and the occurrence of bleeding episodes would help physicians understand the most appropriate way to treat patients, particularly elderly patients. This is critical in Japan where ≥25% of the population is ≥65 years old (2015 census), and those with AF are a little over three‐quarters of a million.^8^, ^9^

Edoxaban is a once‐daily DOAC that directly and reversibly inhibits factor Xa and is indicated for long‐term use in patients with NVAF to prevent ischemic stroke and systemic embolism.^10^, ^11^, ^12^, ^13^ Edoxaban is available in two formulations: tablet and orally disintegrating (OD). Swallowing is a concern for elderly patients, and so the OD formulation is particularly useful because it helps them take the drug daily and consistently in the long‐term. Edoxaban has two more indications: treatment and prevention of recurrence of venous thromboembolism, and prevention of postoperative venous thromboembolism after lower extremity orthopedic surgery.

The efficacy and safety of edoxaban were confirmed in phase‐3 ENGAGE AF‐TIMI‐48;^1^ because it was a pivotal confirmatory study and designed as a randomized controlled trial (RCT), the patient population had strict inclusion and exclusion criteria. Since these studies do not include all patients who would potentially benefit from taking the drug, it is necessary to investigate the safety and effectiveness of edoxaban in a real‐world clinical setting. ETNA‐AF‐Japan (UMIN000017011) was initiated to collect such data over a two‐year period.

We have published three‐month interim analysis results in September 2018^14^ that reported on patient demographics, clinical characteristics, and dosing status. Here, we report on the one‐year interim analysis of data that also includes safety and effectiveness analyses of edoxaban. Furthermore, we analyzed the safety and effectiveness of treatment in patients with a specific background, such as those ≥75 years old, including patients whose body weight is ≤60 kg and who have other factors.

## 2 METHODS

### 2.1 Study design

ETNA‐AF‐Japan is a real‐world, prospective, multicenter observational study that aims to collect the baseline and clinical characteristics of Japanese patients with NVAF and analyze the safety and effectiveness of edoxaban in these patients. This postmarketing surveillance (PMS) was conducted according to the “Good Post‐marketing Study Practice of the Ministry of Health, Labor, and Welfare of Japan.” Detailed methods of this study were published in the three‐month report.^14^

### 2.2 Patient population

Eligible patients were adults with NVAF who were to receive edoxaban for the first time to prevent ischemic stroke and systemic embolism. Further requirements for enrollment were the ability to start treatment during the enrollment period, availability for long‐term follow‐up, and agreement to provide written informed consent to participate at the time of registration.

### 2.3 Survey variables

Physicians recorded, in case report forms, demographic and clinical characteristics and reported the medication status of edoxaban and any other medical treatments or diseases, surgical procedures, or nonpharmacologic treatments for AF that patients had undergone. Patient backgrounds were used to calculate HAS‐BLED,^15^ CHADS_2,_ ^16^ and CHA_2_DS_2_‐VASc^17^ scores. We calculated HAS‐BLED scores according to risk factors of hypertension, abnormal renal function, abnormal liver function, stroke, bleeding, and elderly but excluded the HAS‐BLED score items of the international normalized ratio and alcohol use in the total count.

Critically important clinical events to record were death, stroke other than transient ischemic attack (TIA), systemic embolism, myocardial infarction, and adverse events (AEs), including bleeding events.

### 2.4 Starting daily dose and administration

The recommended daily oral dose, as described in the package insert (PI) in Japan, was 60 mg for adult patients weighing >60 kg and 30 mg for those weighing ≤60.^11^ The 30 mg dose was also recommended for patients with one or more of the following: renal dysfunction (creatinine clearance, CLcr ≤50 mL/min), and concomitant use of the following P‐glycoprotein (P‐gp) inhibitors: quinidine, verapamil, erythromycin, or cyclosporine.

### 2.5 Study outcomes

Study outcomes were recorded as AEs, including bleeding events, and clinical events such as death, stroke, systemic embolism, and myocardial infarction, the latter of which may provide information for estimating the effectiveness of edoxaban. Bleeding events (safety outcomes), ischemic stroke (excluding TIA) and systemic embolism (effectiveness outcomes), were specifically collected and analyzed. Attending physicians categorized the degree of bleeding (major bleeding, clinically relevant non‐major bleeding [CRNMB], and minor bleeding) according to the definitions described in ENGAGE AF‐TIMI 48^18^ with slight modifications (Supplement 1).

### 2.6 Statistical analysis

For categorical variables, proportions were calculated. For continuous variables, summary statistics (mean, SD) were calculated. To determine the one‐year outcomes, we analyzed data up to the 390‐day follow‐up period for each patient. The Kaplan‐Meier Plots were presented for bleeding events and clinical events, respectively. All statistical analyses were performed using SAS System Release 9.2 (SAS Institute Inc).

## 3 RESULTS

### 3.1 Disposition of patients

In total, 11 569 patients with NVAF were enrolled. At one‐year, we had collected case report forms for 11 469 patients and excluded 362 patients (3.2%) for the following reasons: a serious protocol deviation (n = 23), nonimplementation of a safety evaluation (n = 213), consent withdrawal (n = 47), and start of edoxaban treatment with the 15 mg dose (n = 79), a dose not indicated for therapeutic use, resulting in a safety analysis set of 11 107. Forty‐eight patients were excluded for off‐label use for other diseases; thus, the effectiveness analysis set was 11 059 (Flowchart in Supplement 2).

### 3.2 Baseline demographic and clinical characteristics

Table 1 shows the baseline demographic and clinical characteristics and Table 2 shows the patient risk scores of 11 107 patients (safety analysis set). The mean age was 74.2 ± 10.0 years, and 40.6% were female. About half of the patients (52.4%) were ≥75 years old; 54.3% had a body weight ≤60 kg, and 31.2% had a CLcr ≤50 mL/min. A few patients had a history of intracranial (2.3%) or gastrointestinal (1.6%) bleeding, and 20.6% had a history of ischemic stroke or TIA. The mean CHADS_2_ score was 2.2 ± 1.3, the mean CHA_2_DS_2_‐VASc score was 3.5 ± 1.6, and the mean HAS‐BLED score was 2.0 ± 1.0. Overall, 33.9% of patients had a CHADS_2_ score ≤1 (Table 2: score of 0, 8.9%; score of 1, 25.0%). The proportion of patients with high‐risk categories was greater in the 30 mg than in the 60 mg dose group: age ≥75 years (63.4% vs 23.7%); CHADS_2_ score ≥2 (70.2% vs 54.4%), and HAS‐BLED score ≥2 (73.7% vs 58.4%).

**Table 1:** Baseline demographic and clinical characteristics

	Total	Daily dose at the start of the study	
		60 mg	30 mg
	N = 11 107	N = 3066	N = 8041
Female	4508 (40.6)	436 (14.2)	4072 (50.6)
Age, y			
Mean ± SD	74.2 ± 10.0	67.5 ± 9.5	76.7 ± 9.0
≥75	5823 (52.4)	726 (23.7)	5097 (63.4)
Body weight, kg			
Mean ± SD	60.0 ± 12.7	71.5 ± 10.4	55.5 ± 10.6
<40	369 (3.3)	2 (0.1)	367 (4.6)
40 to 60	5662 (51.0)	167 (5.4)	5495 (68.3)
>60	4895 (44.1)	2864 (93.4)	2031 (25.3)
Unknown	181 (1.6)	33 (1.1)	148 (1.8)
Creatinine clearance, mL/min^a^			
Mean ± SD	63.9 ± 25.8	84.8 ± 25.8	55.9 ± 20.8
<30	537 (4.8)	1 (0.0)	536 (6.7)
30 to 50	2928 (26.4)	68 (2.2)	2860 (35.6)
>50 to <80	4979 (44.8)	1410 (46.0)	3569 (44.4)
≥80	2372 (21.4)	1520 (49.6)	852 (10.6)
Unknown	291 (2.6)	67 (2.2)	224 (2.8)
Type of atrial fibrillation			
Paroxysmal	5121 (46.1)	1429 (46.6)	3692 (45.9)
Persistent (>7 days)	4264 (38.4)	1226 (40.0)	3038 (37.8)
Permanent	1706 (15.4)	405 (13.2)	1301(16.2)
Unknown	16 (0.1)	6 (0.2)	10 (0.1)
Switch from other anticoagulants			
Total^b^	2525 (22.7)	681 (22.2)	1844 (22.9)
Warfarin	1237 (11.1)	318 (10.4)	919 (11.4)
Rivaroxaban	445 (4.0)	133 (4.3)	312 (3.9)
Apixaban	362 (3.3)	90 (2.9)	272 (3.4)
Dabigatran	316 (2.8)	97 (3.2)	219 (2.7)
Others	166 (1.5)	44 (1.4)	122 (1.5)
Bleeding history			
Intracranial bleeding	258 (2.3)	56 (1.8)	202 (2.5)
Gastrointestinal bleeding	175 (1.6)	42 (1.4)	133 (1.7)
Medical history/comorbidities			
Hypertension	7991 (71.9)	2231 (72.8)	5760 (71.6)
Diabetes mellitus	2587 (23.3)	801 (26.1)	1786 (22.2)
Dyslipidemia	4020 (36.2)	1235 (40.3)	2785 (34.6)
Myocardial infarction	425 (3.8)	88 (2.9)	337 (4.2)
Angina pectoris	1203 (10.8)	276 (9.0)	927 (11.5)
Heart failure/left ventricular systolic dysfunction	3009 (27.1)	609 (19.9)	2400 (29.8)
Ischemic stroke/transient ischemic attack	2290 (20.6)	568 (18.5)	1722 (21.4)
Cancer	863 (7.8)	195 (6.4)	668 (8.3)
Gastric ulcer	407 (3.7)	103 (3.4)	304 (3.8)
Anemia	452 (4.1)	54 (1.8)	398 (4.9)

Note

Data are presented as number (%) unless otherwise indicated.

Abbreviation: SD, standard deviation.

^a^ Creatinine clearance was estimated using the Cockcroft & Gault equation.

^b^ Some overlap present.

**Table 2:** Patient risk scores

	Total	Starting daily dose	
		60 mg	30 mg
	N = 11 107	N = 3066	N = 8041
CHADS_2_ score			
Mean ± SD	2.2 ± 1.3	1.8 ± 1.3	2.3 ± 1.4
0	993 (8.9)	420 (13.7)	573 (7.1)
1	2777 (25.0)	971 (31.7)	1806 (22.5)
2	3312 (29.8)	881 (28.7)	2431 (30.2)
3	2203 (19.8)	478 (15.6)	1725 (21.5)
4	1150 (10.4)	230 (7.5)	920 (11.4)
5	516 (4.6)	66 (2.2)	450 (5.6)
6	131 (1.2)	14 (0.5)	117 (1.5)
Unknown	25 (0.2)	6 (0.2)	19 (0.2)
CHA_2_DS_2_‐VASc score			
Mean ± SD	3.5 ± 1.6	2.7 ± 1.5	3.8 ± 1.6
0	300 (2.7)	206 (6.7)	94 (1.2)
1	941 (8.5)	514 (16.8)	427 (5.3)
2	1861 (16.8)	751 (24.5)	1110 (13.8)
3	2643 (23.8)	745 (24.3)	1898 (23.6)
4	2453 (22.1)	460 (15.0)	1993 (24.8)
5	1629 (14.7)	262 (8.5)	1367 (17.0)
6	823 (7.4)	92 (3.0)	731 (9.1)
7	341 (3.1)	27 (0.9)	314 (3.9)
8	78 (0.7)	3 (0.1)	75 (0.9)
9	13 (0.1)	0 (0.0)	13 (0.2)
Unknown	25 (0.2)	6 (0.2)	19 (0.2)
HAS‐BLED Score^a^			
Mean ± SD	2.0 ± 1.0	1.7 ± 1.0	2.1 ± 0.9
0	570 (5.1)	316 (10.3)	254 (3.2)
1	2610 (23.5)	908 (29.6)	1702 (21.2)
2	4860 (43.8)	1186 (38.7)	3674 (45.7)
3	2187 (19.7)	478 (15.6)	1709 (21.3)
4	617 (5.6)	119 (3.9)	498 (6.2)
5	53 (0.5)	7 (0.2)	46 (0.6)
6	0 (0.0)	0 (0.0)	0 (0.0)
7	0 (0.0)	0 (0.0)	0 (0.0)
Unknown	210 (1.9)	52 (1.7)	158 (2.0)

Note

Data are presented as number (%) unless otherwise indicated.

Abbreviation: SD, standard deviation.

^a^ Neither labile international normalized ratio nor alcohol use were counted; thus, the highest total score was seven.

### 3.3 Starting daily dose and adjustment factors

**Figure 1:**
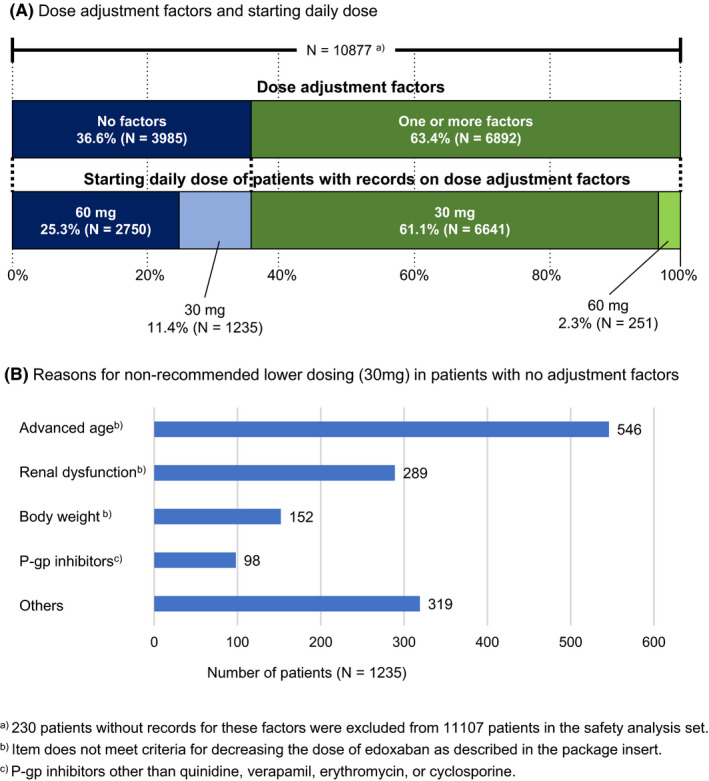
Madication status. (A) dose adjustments, starting dose, and (B) reasons for nonrecommended lower‐dosing. The dose adjustment factors were body weight ≤60 kg, creatinine clearance ≤50 mL/min, or concomitant use of the following P‐glycoprotein (P‐gp) inhibitors: quinidine, verapamil, erythromycin, or cyclosporine

The starting daily dose of edoxaban prescribed to patients in the safety analysis set was 60 mg in 27.6% and 30 mg in 72.4% (Table 1). Of the 11 107 patients, 230 patients without records for dose adjustment factors were excluded. Thus, background information for 10 877 patients was used to determine how edoxaban was used, as described in the PI (Figure 1). Of these 10 877 patients, 9391 (86.3%) received the recommended daily dose of edoxaban: 6641 patients (61.1%) took a 30 mg dose and 2750 patients (25.3%) took a 60 mg dose. The largest group of patients (n = 6641, 61.1%) had dose adjustment factors of low body weight ≤60 kg, CLcr ≤50 mL/min, or concomitant use of a P‐gp inhibitor and took the recommended lower dose of 30 mg. However, 251 patients with adjustment factors (2.3%) started treatment with the 60 mg dose, which is not recommended. Meanwhile, patients without dose adjustment factors (n = 1235, 11.4%) started treatment with the 30 mg dose (nonrecommended lower‐dose).

The reasons physicians gave for prescribing the nonrecommended lower dose were advanced age in 546 patients (44.2%), renal dysfunction in 289 patients (23.4%), and body weight in 152 patients (12.3%). Additional reasons for nonrecommended lower dosing were concomitant use of P‐gp inhibitors other than quinidine, verapamil, erythromycin, or cyclosporine in 98 patients (7.9%). “Others” included patients with bleeding risk (eg, history of bleeding or use of an antithrombotic agent) in 319 patients (25.8%).

### 3.4 Edoxaban treatment status

The mean and median duration of edoxaban treatment during the one‐year follow‐up period was 311.2 ± 118.0 days and 366.0 days (actual treatment period, excluding the stop‐dosing period). As shown in Table 3, edoxaban treatment is ongoing for 8783 patients (79.1%) and was completed or discontinued for 2324 patients (20.9%) for the following reasons: failure to visit the hospital or transfer to a different hospital (n = 826, 7.4%); occurrence of clinical events or AEs (n = 741, 6.7%); treatment completed as planned (n = 350, 3.2%), or switched to other drugs (n = 335, 3.0%). Additional reasons were a plan to receive nonpharmacological therapy for AF (n = 30, 0.3%) and plan to receive an invasive procedure (n = 25, 0.2%).

**Table 3:** Edoxaban treatment status

Total	N = 11 107
Ongoing treatment with edoxaban^a^	8783 (79.1)
Completion or discontinuation of treatment with edoxaban	2324 (20.9)

Reasons for completion or discontinuation of treatment with edoxaban^b^	
Failure to visit the hospital or transfer to a different hospital	826 (7.4)
Occurrence of clinical events or AEs	741 (6.7)
Treatment completed as planned	350 (3.2)
Switch to other drugs	335 (3.0)
Plan to receive nonpharmacological therapy for AF	30 (0.3)
Plan to receive an invasive procedure (including minor surgery)	25 (0.2)

Note

Data are presented as number (%) unless otherwise indicated.

Abbreviations: AE, adverse events; AF, atrial fibrillation.

^a^ Including interruption during one‐year follow‐up

^b^ Some overlap present.

### 3.5 Safety analyses

**Figure 2:**
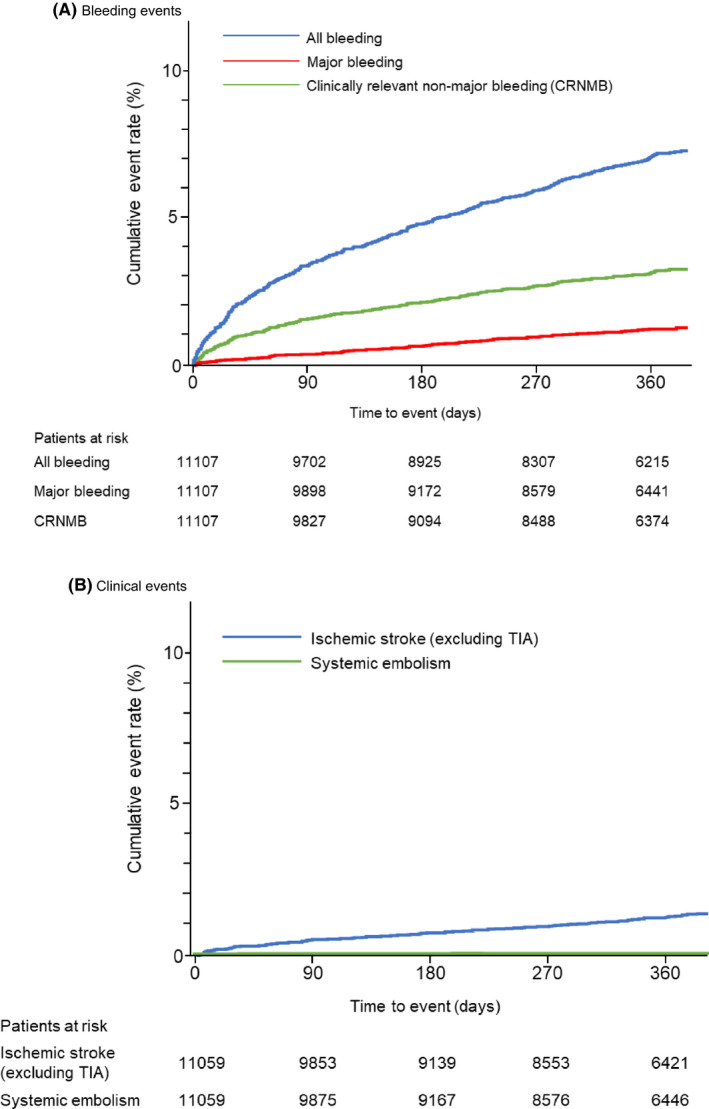
Kaplan‐Meier analyses of (A) bleeding events and (B) clinical events

**Figure 3:**
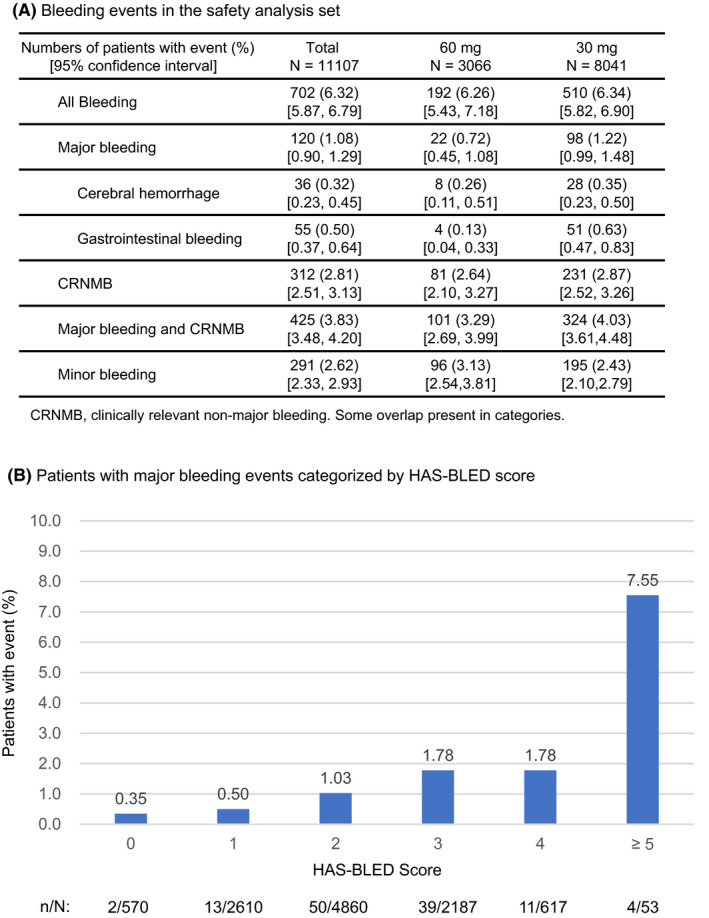
Safety analyses. (A) incidence of bleeding events and (B) incidence of major bleeding events categorized by HAS‐BLED score

The cumulative incidence rate of bleeding events has increased over the year (Figure 2). Figure 3A shows the incidence of bleeding events during the edoxaban treatment period (details in Supplement 3). In the safety analysis set (n = 11 107), the incidence of all bleeding events was 6.32% (n = 702); major bleeding was 1.08% (n = 120), and CRNMB was 2.81% (n = 312). Gastrointestinal bleeding was the most common type (Supplement 3). The incidence of all bleeding events was 6.26% for the 60 mg dose group and 6.34% for 30 mg; for major bleeding, these were 0.72% and 1.22%.

Figure 3B shows the incidence of major bleeding events categorized by HAS‐BLED scores. Patients with scores of 2, 3, or 4 had an incidence of 1.30%, and those with scores of 0 or 1 had an incidence of 0.47%. Patients with a score of ≥5 had an incidence of 7.55% for major bleeding.

### 3.6 Effectiveness analyses

**Figure 4:**
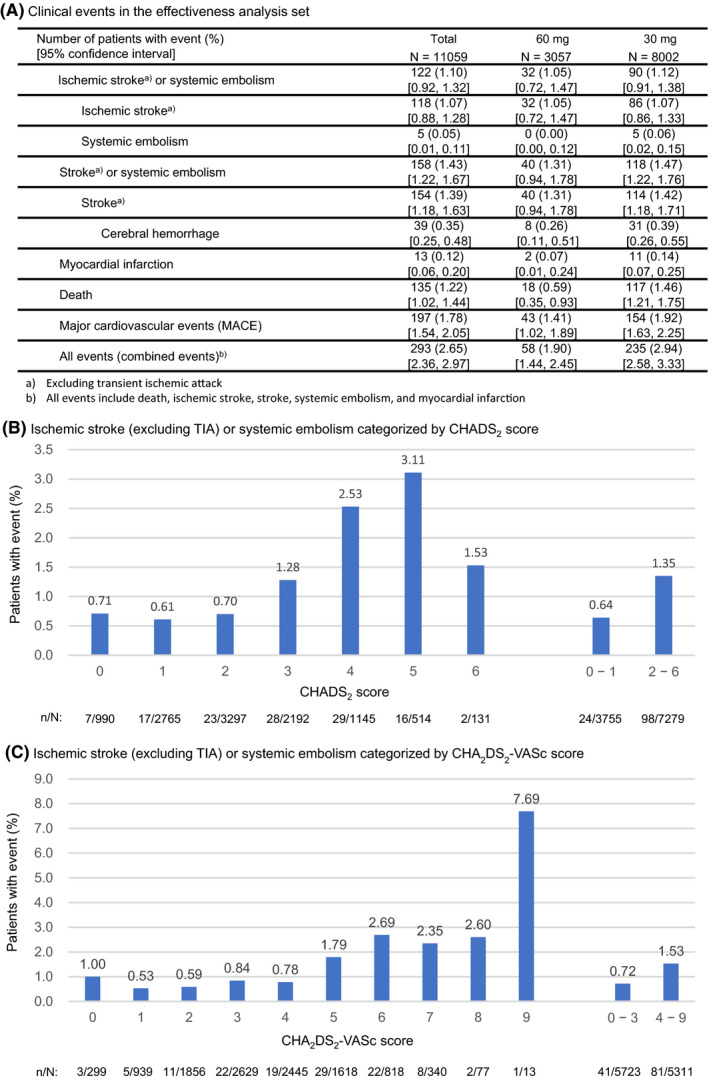
Effectiveness analyses. (A) incidence of clinical events and (B) incidence of ischemic stroke (excluding TIA) or systemic embolism categorized by (B) CHADS_2_ score and (C) CHA_2_DS_2_‐VASc score.

The cumulative incidence rate of clinical events has increased over the year (Figure 2). Figure 4A summarizes the incidence of clinical events during the edoxaban treatment period. In the effectiveness analysis set (n = 11 059), the incidence of thromboembolic events (ischemic stroke [excluding TIA] or systemic embolism) was 1.10% (n = 122): 1.05% (n = 32) at the 60 mg dose and 1.12% (n = 90) at the 30 mg dose.

The incidence of thromboembolic events categorized by CHADS_2_ and CHA_2_DS_2_‐VASc risk scores are shown in Figure 4B,C, respectively. For CHADS_2_, the incidence of thromboembolic events in patients with lower risk scores (0 and 1) was 0.64%, and was 1.35% for those with higher risk scores (≥2). For CHA_2_DS_2_‐VASc, the incidence of events was 0.72% for patients with lower risk scores (0‐3) and was 1.53% for patients with higher risk scores (≥4).

### 3.7 Safety and effectiveness outcomes in patients with a specific background

Table 4 shows the safety and effectiveness of recommended doses of edoxaban treatment in patients with a specific background. Patient groups whose incidence of major bleeding was more than double that of all patients (1.13%) were the dual antiplatelet therapy (DAPT) use group (4.35%) and severely low body weight (<40 kg) group (3.00%). Moreover, patient groups whose incidence of thromboembolic events was more than double that of all patients (1.19%) were the DAPT use group (4.97%), cerebrovascular disease group (2.74%), and antiplatelet agent use group (2.56%).

**Table 4:** Safety and effectiveness outcomes in patients with a specific background

	Safety outcomes: Bleeding events				Effectiveness outcomes	
	All bleeding		Major bleeding		Ischemic stroke (excluding TIA) or systemic embolism	
	(%)	(n/N)	%	(n/N)	%	(n/N)
All patients	6.52	(612/9391)	1.13	(106/9391)	1.19	(111/9351)
Advanced age (≥75 years)	7.83	(391/4996)	1.44	(72/4996)	1.37	(68/4974)
Low body weight (≤60 kg)	6.48	(380/5862)	1.16	(68/5862)	1.13	(66/5833)
Severely low body weight (<40 kg)	9.26	(34/367)	3.00	(11/367)	1.92	(7/364)
CLcr (≤50 mL/min)	7.69	(261/3396)	1.71	(58/3396)	1.48	(50/3377)
Liver dysfunction	9.32	(67/719)	1.53	(11/719)	1.40	(10/714)
Hypertension	6.98	(471/6744)	1.23	(83/6744)	1.24	(83/6719)
Heart disease	8.36	(311/3721)	1.59	(59/3721)	1.19	(44/3690)
Cerebrovascular disease	7.60	(153/2012)	1.89	(38/2012)	2.74	(55/2004)
Cancer	14.25	(104/730)	1.64	(12/730)	1.52	(11/723)
P‐gp inhibitor use^a^	8.07	(51/632)	1.74	(11/632)	1.59	(10/628)
Antiplatelet agent use	11.87	(150/1264)	1.82	(23/1264)	2.56	(32/1252)
DAPT use^b^	21.74	(35/161)	4.35	(7/161)	4.97	(8/161)

^a^ P‐gp inhibitors, quinidine, verapamil, erythromycin, or cyclosporine.

^b^ DAPT, Dual antiplatelet therapy.

## 4 DISCUSSION

ETNA‐AF‐Japan is an observational study of over 10 000 NVAF patients treated with edoxaban. ETNA‐AF‐Japan, a real‐world study in contrast to an RCT such as ENGAGE AF‐TIMI 48, includes various types of patients with multiple risk factors. Therefore, the study could provide information to aid in the physicians’ understanding of bleeding risks and the clinical benefits of anticoagulation with edoxaban.^19^ We report here the one‐year follow‐up on the safety and effectiveness of edoxaban.

### 4.1 Baseline demographics and clinical characteristics

As reported previously,^14^ patient backgrounds in this study were somewhat different from those in the phase‐3 study (ENGAGE AF‐TIMI 48).^1^ ETNA‐AF‐Japan included patients with CHADS_2_ score ≤1, severe renal dysfunction (CLcr <30 mL/min) and DAPT use, which were excluded from ENGAGE AF‐TIMI 48.^1^ Patient backgrounds such as age, body weight, and risk scores in terms of ETNA‐AF‐Japan were similar to those in the real‐world SAKURA AF Registry,^20^, ^21^ Fushimi AF Registry,^22^ and other DOAC observational studies,^23^, ^24^, ^25^, ^26^, ^27^ suggesting that ETNA‐AF‐Japan reflected a real‐world population of Japanese patients with NVAF.

### 4.2 Dose levels and adjustment factors

The lower dose of 30 mg edoxaban was administered to approximately 70% of patients. Of the 10 877 patients, 1235 patients (11.4%) received a nonrecommended lower dose (30 mg). Under‐dosing of DOAC has been reported in many studies. Some studies reported higher thromboembolic events in nonrecommended lower‐dosed patients than in patients with the recommended dose, and others found no significant differences in clinical outcomes between the two patient groups.^20^, ^27^, ^28^, ^29^, ^30^ In this study, 11.4% of patients were receiving a nonrecommended lower‐dose, and physicians might have chosen the dose because of advanced age or renal dysfunction and were considered to be at a higher risk of bleeding. Upon completion of this ongoing two‐year observational study, we expect to provide further information, that would aid physicians in considering better strategies for such patients.

### 4.3 Clinical outcomes (safety and effectiveness)

At one‐year follow‐up, the incidence of all bleeding events was 6.32%, and was 1.08% for major bleeding. Although a direct comparison is inappropriate, the incidence of major bleeding was lower in the present study than in ENGAGE AF‐TIMI 48 overall data (2.75% of patients/year)^1^ and in ENGAGE AF‐TIMI 48 Japanese patient data (3.38% of patients/year).^31^ Major bleeding events were mostly gastrointestinal bleeding, which was similar to that of ENGAGE AF‐TIMI 48. In the SAKURA AF Registry^20^ and Fushimi AF Registry,^22^ the incidence rate of major bleeding was 1.21% and 1.8% of patients/year, respectively. Although these observational studies differ from ETNA‐AF‐Japan in terms of the follow‐up period and drugs prescribed, the incidence of major bleeding in ETNA‐AF‐Japan (1.08%) was not higher than those in other registries. At one‐year follow‐up, the incidence of clinical events was 1.10% for thromboembolic events (ischemic stroke [excluding TIA] or systemic embolism) and 1.43% for stroke (excluding TIA) or systemic embolism. It was similar to incidences reported in ENGAGE AF‐TIMI 48 (1.18% of patients/year)^1^ and in ENGAGE AF‐TIMI 48 patients in Japan (1.47% of patients/year).^31^ Moreover, the incidence was not higher than that reported for patients in the SAKURA AF Registry (1.47% of patients/year) and in the Fushimi AF Registry (2.3% of patients/year). The results of ETNA‐AF‐Japan suggested that edoxaban has a clinically acceptable profile in real‐world clinical settings during one‐year treatment.

### 4.4 Safety and effectiveness in the 60 mg and 30 mg groups

The mean HAS‐BLED, CHADS_2_, and CHA_2_DS_2_‐VASc score in the 30 mg group (2.1 ± 0.9, 2.3 ± 1.4 and 3.8 ± 1.6) were higher than those of the 60 mg group (1.7 ± 1.0, 1.8 ± 1.3 and 2.7 ± 1.5). These results suggest that the lower dose (30 mg) was given not only to patients at high risk of bleeding but also to patients at high risk of stroke. Thus, the safety and effectiveness results for the 30 mg group are particularly important. The incidence of all bleeding in the 30 mg (6.34%) and 60 mg (6.26%) groups were similar, and the incidence of major bleeding in the 30 mg (1.22%) was higher than 60 mg (0.72%) group. However, the incidence of major bleeding in the 30 mg group (1.22%) was not higher than that in other real‐world studies (SAKURA AF Registry, 1.21% of patients/year; Fushimi AF Registry, 1.8% of patients/year). The incidence of thromboembolic events was similar in the 30 mg (1.12%) and 60 mg (1.05%) groups. Therefore, these preliminary, one‐year results suggested that there are no major concerns about the safety and effectiveness of edoxaban at the lower dose in a real‐world clinical setting.

### 4.5 Safety and effectiveness outcomes in patients with a specific background

Patients with a specific background (eg, low body weight, patients with renal impairment, or cancer) are not sufficiently included in pivotal clinical trials but are more likely to be included in large‐scale observational studies in the real world. It is important to provide information about the safety and effectiveness profile of these patients, which may be helpful for physicians in clinical practice. The incidence of major bleeding was more than double in patients who had factors of DAPT use and severely low body weight than in all patients in the study. In the phase 3 study, patients using DAPT were excluded, and information on low‐weight patients weighing <40 kg was limited. However, the results of this study point to a higher incidence of major bleeding in these patient groups. The factors for which the incidence of thromboembolic events was more than double that of all patients in the study were history of cerebrovascular disease, antiplatelet agent use, and DAPT use. Cerebrovascular disease includes a history of ischemic stroke, which had a high probability of recurrence. Moreover since an antiplatelet agent or DAPT is administered to patients who are at a high risk of a thromboembolic event, it is expected that incidence rates for these patients would be high. Therefore, it becomes necessary to monitor patients carefully for bleeding or thromboembolic events in the presence of the following factors: severely low body weight, cerebrovascular disease, antiplatelet agent use, or DAPT use.

### 4.6 Limitations

First, the present study was an open‐label observational study with no comparative arm. Therefore, it is not possible to compare edoxaban treatment with other DOACs, or a vitamin K anticoagulant such as warfarin. Second, this ongoing study is an observational study to analyze case report forms provided by physicians. As is natural in any clinical setting, some patients did not return for follow‐up visits. The loss of patients to follow‐up might have led to an underestimation of the event rates. Third, the one‐year analysis of data reported here are interim results; since the follow‐up is ongoing, a statistical analysis should wait until the final report. Therefore, it is challenging to compare one‐year of accumulated data with studies that have continued for three or more years, which has made comparisons with these other studies somewhat arbitrary. However, these comparisons are useful because we were able to observe general trends about what we should be vigilant about, such as the monitoring of high‐risk patients, particularly those who are being treated with multiple drugs for serious illnesses.

## 5 CONCLUSIONS

This one‐year interim analysis of 11 107 patients in the ETNA‐AF‐Japan study provided real‐world information about Japanese patients with NVAF and data on edoxaban use in high‐risk patients who are commonly excluded in phase‐3 studies. The results suggested that there were no major concerns about the safety and effectiveness of edoxaban use in daily clinical practice in Japan at one‐year follow‐up. After completion of the two‐year observation analysis, we will have a clearer picture of edoxaban use in clinical practice and further understand the safety and effectiveness profiles of edoxaban.

## CONFLICT OF INTEREST

TY received consulting and lecture fees from Daiichi Sankyo Co. Ltd., Bayer Yakuhin Ltd., Bristol Myers Squibb, Ono Pharmaceutical Co. Ltd., Toa Eiyo Ltd., and Boehringer Ingelheim Japan Co. Ltd. and research funding from Daiichi Sankyo Co. Ltd., Bayer Yakuhin Ltd., and Bristol Myers Squibb. YK received consulting and lecture fees from Daiichi Sankyo Co. Ltd., Bayer Yakuhin Ltd., Bristol Myers Squibb, and Boehringer Ingelheim Japan Co. Ltd. TN and KS are employees of Daiichi Sankyo Co. Ltd.

## Supporting information

Supplementary Material

Supplementary Material

Supplementary Material
