# Supplementary material for: Postmarketing surveillance on the clinical use of edoxaban in patients with nonvalvular atrial fibrillation (ETNA‐AF‐Japan): One‐year safety and effectiveness analyses
Source: J Arrhythm. 2020 Mar 24;36(3):395–405. doi: 10.1002/joa3.12332 (PMC7279995; doi:10.1002/joa3.12332)
Supplement: Supplementary file 1 — Supplementary Material [file JOA3-36-395-s001.pdf]

Supplement 1. Definitions of bleeding[18]

Major bleeding event
A major bleeding event is defined as a clinically overt bleeding event (i.e., bleeding that is visualized by examination or radiographic imaging) that meets ≥1 of the following.<br>・ Fatal bleeding<br>・ Symptomatic bleeding in a critical area or organ such as retroperitoneal, intracranial, intraocular, intraspinal, intraarticular, pericardial, and intramuscular with compartment syndrome.<br>・ A clinically overt bleeding event that causes a fall in hemoglobin level of ≥2.0 g/dL, adjusted for transfusions. Each 2 units of packed red blood cells or whole blood (400 mL) is counted as a 1.0 g/dL decrease in hemoglobin. In the case of surgical procedural-related bleeding, the bleeding must be in excess of that normally associated with the surgery/procedure. In the absence of hemoglobin data, a fall of hematocrit of ≥6.0%, adjusted for transfusion, will satisfy the criteria for a major bleeding event.
Clinically relevant non-major bleeding
A clinically relevant non-major bleeding event is defined as a clinically overt bleeding event that requires medical attention. Examples of bleeding requiring medical attention include, but are not limited to, bleeding events that result in the following diagnostic or therapeutic measures:<br>・ Requires or prolongs hospitalization<br>・ Laboratory evaluation<br>・ Imaging studies<br>・ Endoscopy, colonoscopy, cystoscopy, or bronchoscopy<br>・ Nasal packing<br>・ Compression<br>・ Ultrasound-guided closure of an aneurysm<br>・ Coil embolization<br>・ Inotropic support<br>・ Surgery<br>・ Interruption or stopping study medication at the advice of a healthcare provider<br>・ Changing concomitant therapies (eg, reducing the dose or discontinuing aspirin at the advice of a healthcare provider<br>Note: An outpatient visit without any of the above or similar diagnostic/therapeutic measures does not satisfy the criteria for “requiring medical attention.”
Minor (non clinically relevant) bleeding
Other overt bleeding events that do not fulfill the criteria of a major bleeding event or a clinically relevant nonmajor bleeding event (eg, epistaxis that does not require medical attention) will be classified as minor bleeding events.<br>Note: All other events (e.g., decline in hemoglobin with no overt bleeding event) will be classified as “non-bleeding events.”
