# Supplementary figures and images for: Postmarketing surveillance on the clinical use of edoxaban in patients with nonvalvular atrial fibrillation (ETNA‐AF‐Japan): One‐year safety and effectiveness analyses
Source: J Arrhythm. 2020 Mar 24;36(3):395–405. doi: 10.1002/joa3.12332 (PMC7279995; doi:10.1002/joa3.12332)

Supplement 2. Patient disposition of edoxaban administration

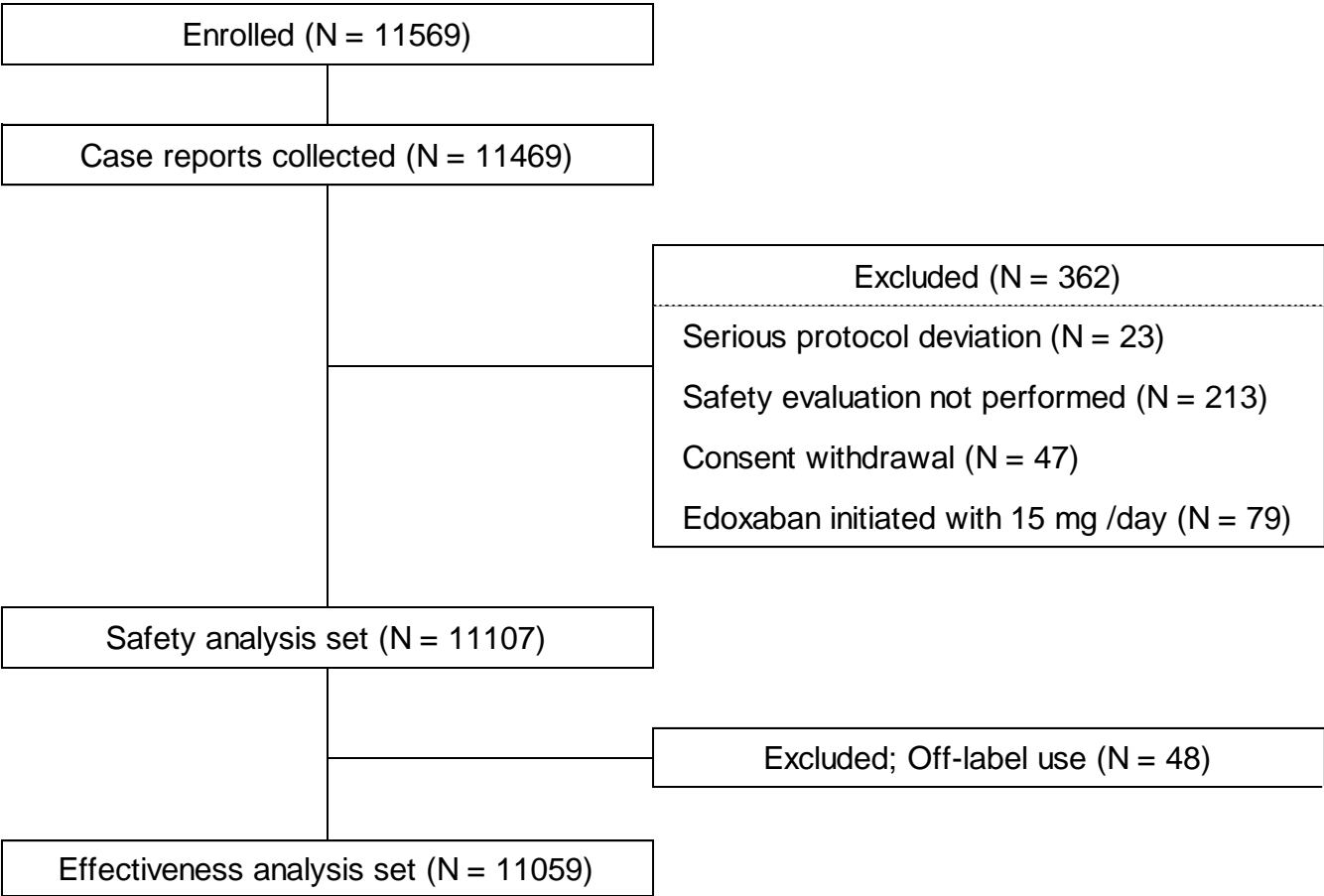

Supplement: Supplementary file 2 — Supplementary Material [file JOA3-36-395-s002.pdf]
