# Supplementary material for: Postmarketing surveillance on the clinical use of edoxaban in patients with nonvalvular atrial fibrillation (ETNA‐AF‐Japan): One‐year safety and effectiveness analyses
Source: J Arrhythm. 2020 Mar 24;36(3):395–405. doi: 10.1002/joa3.12332 (PMC7279995; doi:10.1002/joa3.12332)
Supplement: Supplementary file 3 — Supplementary Material [file JOA3-36-395-s003.pdf]

Supplement 3. Major bleeding events summary

Details of major bleeding events during the edoxaban treatment period in the safety analysis set (N=11107)

	Total (N = 11107)	60 mg (N = 3066)	30 mg (N = 8041)
Total no. of patients, n (%)	120 (1.08)	22 (0.72)	98 (1.22)
Total no. of events	125	23	102
Neoplasms benign, malignant and unspecified (incl cysts and polyps)	2 (0.02)	1 (0.03)	1 (0.01)
Tumour haemorrhage	2 (0.02)	1 (0.03)	1 (0.01)
Blood and lymphatic system disorders	2 (0.02)	0 (0.00)	2 (0.02)
Anaemia	1 (0.01)	0 (0.00)	1 (0.01)
Iron deficiency anaemia	1 (0.01)	0 (0.00)	1 (0.01)
Haemorrhagic diathesis	0 (0.00)	0 (0.00)	0 (0.00)
Nervous system disorders	37 (0.33)	8 (0.26)	29 (0.36)
Brain stem haemorrhage	1 (0.01)	0 (0.00)	1 (0.01)
Cerebellar haemorrhage	6 (0.05)	1 (0.03)	5 (0.06)
Cerebral haemorrhage	17 (0.15)	4 (0.13)	13 (0.16)
Haemorrhage intracranial	1 (0.01)	0 (0.00)	1 (0.01)
Subarachnoid haemorrhage	6 (0.05)	1 (0.03)	5 (0.06)
Thalamus haemorrhage	3 (0.03)	1 (0.03)	2 (0.02)
Putamen haemorrhage	3 (0.03)	1 (0.03)	2 (0.02)
Intracranial haematoma	1 (0.01)	0 (0.00)	1 (0.01)
Eye disorders	2 (0.02)	0 (0.00)	2 (0.02)
Retinal haemorrhage	2 (0.02)	0 (0.00)	2 (0.02)
Cardiac disorders	4 (0.04)	2 (0.07)	2 (0.02)
Cardiac tamponade	4 (0.04)	2 (0.07)	2 (0.02)
Vascular disorders	6 (0.05)	1 (0.03)	5 (0.06)
Aortic aneurysm rupture	5 (0.05)	1 (0.03)	4 (0.05)
Haematoma	1 (0.01)	0 (0.00)	1 (0.01)
Respiratory, thoracic and mediastinal disorders	3 (0.03)	2 (0.07)	1 (0.01)
Epistaxis	1 (0.01)	1 (0.03)	0 (0.00)
Pulmonary alveolar haemorrhage	2 (0.02)	1 (0.03)	1 (0.01)
Gastrointestinal disorders	55 (0.50)	4 (0.13)	51 (0.63)
Diverticulum intestinal haemorrhagic	3 (0.03)	1 (0.03)	2 (0.02)
Duodenal ulcer haemorrhage	1 (0.01)	0 (0.00)	1 (0.01)
Faeces discoloured	1 (0.01)	0 (0.00)	1 (0.01)
Gastric haemorrhage	8 (0.07)	1 (0.03)	7 (0.09)
Gastric ulcer haemorrhage	3 (0.03)	0 (0.00)	3 (0.04)
Gastritis haemorrhagic	1 (0.01)	0 (0.00)	1 (0.01)
Gastrointestinal haemorrhage	21 (0.19)	2 (0.07)	19 (0.24)
Haematochezia	1 (0.01)	0 (0.00)	1 (0.01)
Melaena	3 (0.03)	0 (0.00)	3 (0.04)
Rectal haemorrhage	1 (0.01)	0 (0.00)	1 (0.01)
Upper gastrointestinal haemorrhage	3 (0.03)	0 (0.00)	3 (0.04)
Lower gastrointestinal haemorrhage	7 (0.06)	0 (0.00)	7 (0.09)
Large intestinal haemorrhage	2 (0.02)	0 (0.00)	2 (0.02)

	Total (N = 11107)	60 mg (N = 3066)	30 mg (N = 8041)
Small intestinal haemorrhage	1 (0.01)	0 (0.00)	1 (0.01)
Gastrointestinal angiectasia	1 (0.01)	0 (0.00)	1 (0.01)
Musculoskeletal and connective tissue disorders	2 (0.02)	1 (0.03)	1 (0.01)
Haemarthrosis	1 (0.01)	0 (0.00)	1 (0.01)
Muscle haemorrhage	1 (0.01)	1 (0.03)	0 (0.00)
Renal and urinary disorders	1 (0.01)	1 (0.03)	0 (0.00)
Renal haemorrhage	1 (0.01)	1 (0.03)	0 (0.00)
Injury, poisoning and procedural complications	8 (0.07)	3 (0.10)	5 (0.06)
Subdural haematoma	5 (0.05)	2 (0.07)	3 (0.04)
Subdural haemorrhage	2 (0.02)	1 (0.03)	1 (0.01)
Post procedural haemorrhage	0 (0.00)	0 (0.00)	0 (0.00)
Procedural haemorrhage	1 (0.01)	0 (0.00)	1 (0.01)

System organ classes were used in the calculation of the number of patients with AEs, and preferred terms were used in the calculation of the number of AEs (MedDRA/J version 22.0).
